# Development and Characterization of a Parallelizable Perfusion Bioreactor for 3D Cell Culture

**DOI:** 10.3390/bioengineering4020051

**Published:** 2017-05-25

**Authors:** Dominik Egger, Monica Fischer, Andreas Clementi, Volker Ribitsch, Jan Hansmann, Cornelia Kasper

**Affiliations:** 1 Department of Biotechnology, University of Natural Resources and Life Sciences, Muthgasse 18, 1190 Vienna, Austria; dominik.egger@boku.ac.at (D.E.); monica.fischer@gmx.at (M.F.); andreas.clementi@boku.ac.at (A.C.); 2 Institute of Chemistry, University of Graz, Heinrichstraße 28/IV, 8010 Graz, Austria; volker.ribitsch@uni-graz.at; 3 Translational Center, University Hospital Wurzburg, Roentgenring 11, 97070 Wuerzburg, Germany; jan.hansmann@uni-wuerzburg.de

**Keywords:** perfusion bioreactor system, 3D cell culture, dynamic cultivation, fluid shear stress, computational fluid dynamics

## Abstract

The three dimensional (3D) cultivation of stem cells in dynamic bioreactor systems is essential in the context of regenerative medicine. Still, there is a lack of bioreactor systems that allow the cultivation of multiple independent samples under different conditions while ensuring comprehensive control over the mechanical environment. Therefore, we developed a miniaturized, parallelizable perfusion bioreactor system with two different bioreactor chambers. Pressure sensors were also implemented to determine the permeability of biomaterials which allows us to approximate the shear stress conditions. To characterize the flow velocity and shear stress profile of a porous scaffold in both bioreactor chambers, a computational fluid dynamics analysis was performed. Furthermore, the mixing behavior was characterized by acquisition of the residence time distributions. Finally, the effects of the different flow and shear stress profiles of the bioreactor chambers on osteogenic differentiation of human mesenchymal stem cells were evaluated in a proof of concept study. In conclusion, the data from computational fluid dynamics and shear stress calculations were found to be predictable for relative comparison of the bioreactor geometries, but not for final determination of the optimal flow rate. However, we suggest that the system is beneficial for parallel dynamic cultivation of multiple samples for 3D cell culture processes.

## 1. Introduction

During the last decade, the importance of three dimensional (3D) cultivation of stem cells in dynamic bioreactor systems for tissue engineering processes, biomaterial testing, and in vitro models became very important. Conventional two dimensional (2D) static cultivation systems are used in many studies, although they do not represent the in vivo situation. Moreover, static systems have disadvantages in the mass transport of nutrients and oxygen into 3D constructs [1]. To overcome these drawbacks, different bioreactors have been developed ranging from spinner flasks [2,3,4], stirred systems [5,6], rotating wall [7] and rotating bed [8,9], to perfusion bioreactors [10,11,12,13] and also microfluidic systems [14,15].

While spinner flasks, stirred systems, and rotating wall reactors provide for a homogeneous distribution of nutrients in the bioreactor chamber, the mass transport into a 3D cell-scaffold construct is limited. In contrast, perfusion bioreactors force the fluid to actively pass through the scaffold in order to enhance mass transport and avoid concentration gradients [13]. Moreover, they can be used to control the mechanical environment via the application of fluid shear forces or hydrostatic pressure [16,17]. Several perfusion systems have been developed and proved to be beneficial for various 3D cell culture purposes [18,19,20,21]. However, currently available systems often have limitations and drawbacks. Most of the systems lack the possibility to cultivate multiple independent samples at once under different conditions. Therefore, the optimization of cell culture conditions is time consuming, costly, and the results may not be reproducible. Online monitoring and recording of cell culture parameters (O_2_, CO_2_, and temperature) is often neglected and valuable information is not available. In addition, most of the systems are not flexible with regards to scaffold size and stiffness, bioreactor chamber and tubing material, or programming of different flow regimes (e.g., alternating or intermittent flow regimes).

Mechanical stimuli such as compression, tension, hydrostatic pressure, or fluid shear forces influence stem cell behavior and often support differentiation towards a specific lineage [16,17,22]. Consequently, comprehensive knowledge and control over the fluid shear forces in a perfusion bioreactor is desirable. When the bioreactor system and material is well characterized and thus inherent parameters are known, integrated pressure sensors can be used to estimate and control fluid shear forces during cultivation.

Thus, in this study we developed a miniaturized perfusion bioreactor system that together with a tailor-made incubator system is flexible, modular, and parallelizable (up to 16 bioreactors). While a previous study focused on the application of this system [23], this study concentrates on the technical characterization. Implemented pressure sensors were characterized and used to determine the permeability of a porous scaffold which is an important material characteristic to predict shear stress. Furthermore, two bioreactor chambers with different geometries were designed and manufactured. Computational simulations were conducted together with pressure measurements and wash out experiments to characterize and compare the bioreactors for their flow velocity profile, residence time distribution, and shear stress conditions. In a proof of concept study, both bioreactor chambers were compared and the entire system was investigated for its suitability for a bone tissue engineering process. For this, human adipose derived mesenchymal stem cells (ASCs) were seeded on the previously characterized porous scaffold and cultivated for 21 days. The flow rate was set to generate shear stress conditions in a physiologic range according to prior shear stress estimations by mathematical models and CFD simulations of both bioreactor chambers.

## 2. Materials and Methods

### 2.1. Bioreactor Design

**Figure 1:**
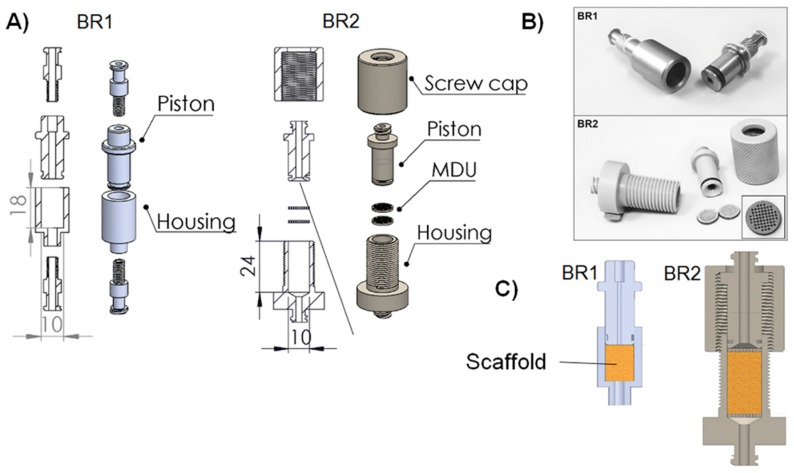
(**A**) Explosion view, (**B**) cross section with inserted biomaterial and (**C**) pictures of the bioreactor chamber prototypes. The first prototype (BR1) was made of stainless steel whereas the second prototype (BR2) made of polyoxymethylene (POM) features two sieve-like medium distribution units (MDU). The flow channel of BR2 opens up to the full diameter of the chamber (detailed view) and the chamber is adjustable in height to allow scaffolds of different thickness to be inserted. The placement of the scaffold in both bioreactors is represented with a yellow box in panel B.

For the development of a perfusion bioreactor chamber, two different prototypes were designed with the help of computer aided design (CAD). Both the chambers were shaped to be suitable for biomaterials that were 10 mm in diameter (Figure 1). The first bioreactor (BR1) was constructed as simply as possible consisting of only a piston, a housing, and two Luer lock screws made from stainless steel, as described before [24]. The inner flow channel of the piston was set to a diameter of 3 mm and the maximum scaffold thickness was limited to 12 mm. A fluorelastomer tubing system (VWR, Darmstadt, Germany) with an inner diameter of 1.6 mm connected the bioreactor chamber to a medium reservoir via Luer lock connectors (Pieper Filter, Bad Zwischenahn, Germany). Sealing rings made of ethylene propylene diene monomer (EPDM) 70 (COG, Pinneberg, Germany) were used for sealing of the inner part and the Luer lock connections.

With the second prototype (BR2), several improvements were implemented. Sieve-like medium distribution units (MDU) hold the matrices in place and support proper medium distribution. To force the liquid to flow through the MDUs, they are 10 mm in diameter and sealed with an EPDM 70 ring. Furthermore, 46 pores with a diameter of 650 µm are arranged in a rectangular grid.

Upstream of the first MDU, the flow channel of the piston opens up to the full diameter of the chamber (detailed view, Figure 1) to support a more homogeneous flow through the matrix and avoid dead spaces. Downstream of the second MDU, the channel narrows again. Besides, a screw cap was introduced to make the bioreactor chamber more flexible with regards to the thickness of the scaffolds inserted. Matrices with a thickness of 18 mm can be inserted into BR2 while the MDUs hold them in place. Additionally, the Luer lock connectors were implemented in the piston and housing to reduce possible leakage sites. In contrast to BR1, BR2 and the MDUs were manufactured from polyoxymethylene (POM). Materials for both bioreactors were chosen to be compatible with steam sterilization.

### 2.2. Incubator System

**Figure 2:**
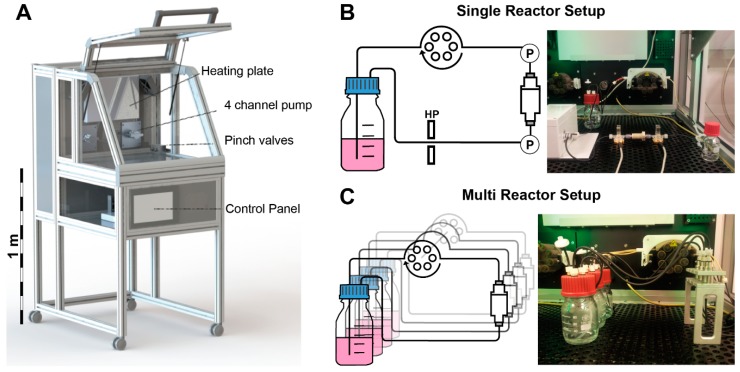
(**A**) Tailor made incubator system developed by Fraunhofer IGB, Stuttgart. A heating plate controls the temperature of the incubator chamber. Two 4 channel pumps can operate several tubing systems at once. A pinch valve can be used to apply hydrostatic pressure (HP). All functions are controlled via a touch screen control panel and all data can be recorded via USB port; (**B**) Single reactor setup in the incubator system: pressure sensors (P) measure the pressure differential inside the bioreactor system non-invasively; (**C**) Multi reactor setup: up to eight independent bioreactors can be operated in parallel (modified from [23], with permission from S. Karger AG, Medical and Scientific Publishers).

The incubator system “Incubator S 2220” was developed, manufactured, and modified by Fraunhofer IGB, Stuttgart, Germany. This custom-made system is equipped with two peristaltic pumps with a 4-channel pump head (ISM 915 and ISM 721 from ISMATEC, Wertheim, Germany) and two pressure sensors (SP 844 from MEMSCAP, Durham, NC, USA). All standard cell culture parameters like temperature, ambient CO_2_, and O_2_ are controlled by a Siemens SIMATIC controller. The incubator chamber is easily accessible and allows safe handling of the bioreactor parts. All parameters are monitored and controlled via integrated touch screen panel (Figure 2). The pumping speed is manually adjustable or can be controlled by a feedback loop with the pressure sensors. The incubator chamber can be either equipped with a single bioreactor system, which is equipped with two pressure sensors, an O_2_-sensor, and a pinch valve for the application of hydrostatic pressure, or with up to eight separate bioreactor systems without additional sensors. Both the setups feature independent media circuits.

### 2.3. Pressure Sensor Characterization

**Figure 3:**
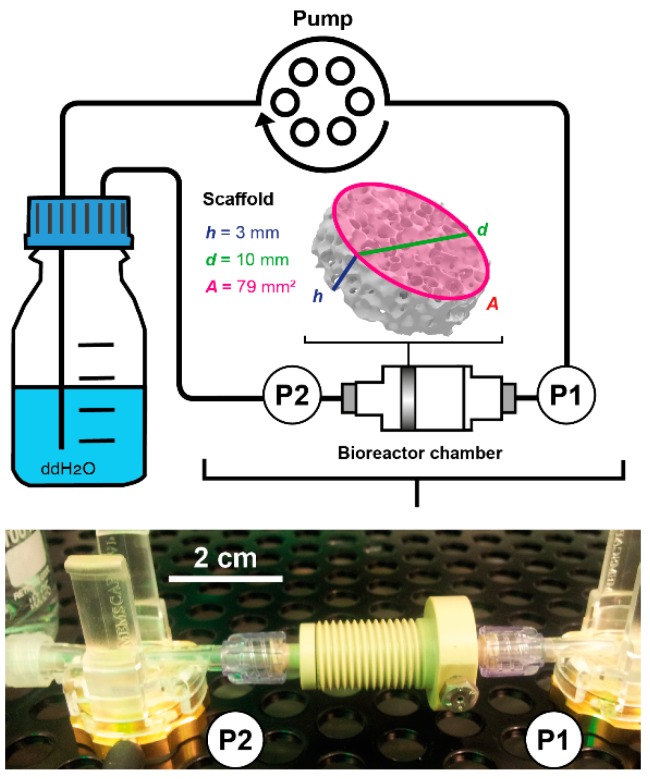
Scheme and picture of the bioreactor setup used during fluid shear stress prediction measurements. Water at 37 °C was pumped through the bioreactor chamber containing the porous scaffold Sponceram. Pressure sensors measured the pressure upstream (P1) and downstream (P2) of the bioreactor chamber non-invasively. Sponceram is depicted as volume rendering of a microCT scan and scaffold dimensions are given as *h* = height, *d* = diameter, and *A* = area.

The pressure in the bioreactor tubing system was studied for each sensor separately at flow rates ranging from 1.5 to 15 mL/min. For this, the pump was programmed to increase the flow rate stepwise by 0.5 mL/min every 10 min (*n* = 3). As the data acquisition system recorded each change in pressure, these measurements resulted in 4423 ± 4 data points for each flow rate. A circular bioreactor setup filled with double distilled water (37 °C) was used during sensor characterization (Figure 3).

### 2.4. Determination of Permeability

The permeability *k* is an important material constant, which allows the estimation of shear forces occurring at different flow velocities. To measure the permeability of a porous scaffold, the pressure inside the bioreactor system was measured simultaneously upstream (P_1_) and downstream of the bioreactor chamber (P_2_) at different flow rates (Figure 3). The differential pressure Δ*P* (P_1_−P_2_) can be used together with characteristics of the biomaterial to calculate the permeability *k* with Darcy’s law:

(1) $$k=\frac{Q\cdot µ\cdot h}{A\cdot \Delta P}$$

where *Q* refers to the volumetric flow rate, *µ* is the dynamic viscosity of water at 37 °C, *h* is the height, and *A* the area of the biomaterial. The scaffold Sponceram (Zellwerk GmbH, Eichstaedt, Germany) used in this study is a ceramic zirconium dioxide matrix (Figure 3). Porosity (66.7%) and average pore size (510 µm) were derived from µCT scans (data not shown). It was shown to have bone-like properties [25] and thus was suggested to be a suitable matrix for bone tissue engineering processes. The scaffold discs used in this study were 10 mm in diameter and 3 mm in thickness.

The differential pressure was measured at different flow rates ranging from 1.5 to 15 mL/min (increment of 0.5 mL/min) with the same setup used during sensor characterization (Figure 3). The data was recorded with the incubator’s data acquisition. Each flow rate was measured for 10 min resulting in 4484 ± 71 data points for each flow rate and measurement. Three randomly picked Sponceram discs were used for the measurements (each *n* = 3).

### 2.5. Computational Fluid Dynamics

To estimate the flow profile and streamlines in the empty bioreactor, 3D models of both bioreactor cartridges were generated with CAD in Solidworks 2015 (Waltham, MA, USA) and imported to COMSOL Multiphysics™ (Burlington, Florence, NJ, USA). The porous media flow model was used where the steady-state Navier-Stokes equations were solved. The material was set to *water* at 37 °C, the inlet boundary condition to *velocity* with *normal inflow velocity*, and the outlet boundary condition was set to *pressure*. For all solid walls, *no slip boundary* conditions were set. The mesh was created by COMSOL with a *normal* element size. The shear stress distribution in a porous scaffold was simulated with the same model. To simplify the scaffold, a cylinder of the same dimensions with porous matrix conditions was introduced to the model. The porosity of the scaffold was set to be 66.7% and permeability was determined by measuring the pressure differential of the bioreactor in- and outlet and was set to 1.7 ± 0.9 × 10^−10^ m^2^ (see Section 2.4).

A stationary study was conducted using 3.6, 17.8, and 35.6 mm/s as flow velocities at the bioreactor inlet which corresponds to the volumetric flow rates of 1.5, 7.5, and 15 mL/min. The velocity profile, streamlines, and shear stress distribution of the bioreactor chambers were plotted from these results.

### 2.6. Residence Time Distribution

To characterize the mixing behavior of a bioreactor system, the residence time distribution (RTD) can be obtained from wash out experiments. For this, a Dirac pulse with the tracer substance methylene blue is injected at the entrance of the bioreactor chamber. Simultaneously, the concentration of the tracer substance at the exit is measured.

First, the bioreactor volume *V_R_* was measured by weighing the reactor chamber with and without water. The volumetric flow rate *Q* was measured by weighing the water that was pumped through the system within a certain time. The hydrodynamic residence time *T* was derived from *V_R_*/*Q*.

To obtain the RTD, a Dirac pulse of 100 µL methylene blue solution (Carl Roth, Karlsruhe, Germany) 1:18 in ddH_2_O was injected at the entrance of the bioreactor chamber during perfusion. Drops were collected 10 cm downstream in a 96 well plate and the absorbance at 688 nm of each well was measured with a plate reader (Infinite M1000, Tecan, Männedorf, Switzerland). The measurement was carried out for *t* = 0... 4 × *T* at 0.6, 1.5, and 3 mL/min (at least *n* = 3). To investigate the influence of a bone like porous matrix inside the chamber, the measurements were performed with and without Sponceram.

The RTD was then derived from the data collected in the washout experiments as described before [9]. Briefly, the residence time function *E*(*t*) is calculated by dividing the concentration of the tracer at each time point by the integral of the tracer concentration from 0 to 4 × *T*. To compare measurements of different bioreactors, the dimensionless residence time function E(Θ) can be derived from *E*(*t*).

The tanks-in-series (TIS) model describes real bioreactors as a cascade of perfectly mixed continuous stirred tank reactors (CSTR) with *N* tanks in series [26]. For *N*→1 the bioreactor behaves like a CSTR, and for *N*→∞ it behaves like a plug flow reactor (PFR) without any axial mixing. Data obtained in the washout experiments was fit to the TIS model with a global curve fit using the software OriginPro (OriginLab, Northampton, MA, USA) with *N* as the key parameter of the following equation:

(2) $$E\left(\Theta \right)=\frac{N{\left(N\Theta \right)}^{N-1}}{\left(N-1\right)!}\;e^{\left(-N\Theta \right)}$$

A real bioreactor can also be described with the dispersion model where the dimensionless Bodenstein number *Bo* describes the ratio between convective transport and axial diffusion. For a system with open-open boundary conditions it can be derived from the response curve of a Dirac pulse as follows:

(3) $$Bo=\frac{1+\sqrt{8\cdot \sigma_{\Theta }^{2}+1}}{\sigma_{\Theta }^{2}}$$

(4) $$\sigma_{\Theta }^{2}=\frac{\sigma^{2}}{T^{2}}$$

With $\sigma^{2}$ as the variance and $\sigma_{\Theta }^{2}$ as the dimensionless variance. For *Bo*→0 the axial dispersion is high, indicating strong back mixing. For *Bo*→∞, the axial dispersion is 0, indicating no back mixing.

### 2.7. Fluid Shear Stress Estimation

Fluid flow induced shear stress is an important parameter in cell culture processes. Thus, after determination of the permeability, the fluid shear stress was calculated from the CFD data. For laminar flow systems, the wall shear stress *τ_ω_* is defined by the normal velocity gradient at the wall:

(5) $$\tau_{\omega }=µ\frac{\partial u}{\partial n}$$

where *µ* is the dynamic viscosity, *u* the flow velocity, and *n* is the *x*-, *y*-, and *z*-direction. Based on Equation (5), the average ($\tau_{\omega avrg}$) and maximum shear stress ($\tau_{\omega max}$) was calculated from the entire scaffold *domain* which was introduced in the COMSOL model as described in Section 2.5. Furthermore, the CFD derived shear stress was compared with a model proposed by Vossenberg et al. [27] which uses the permeability constant k as an indicator for shear stress.

It also uses the permeability constant *k* to calculate $\tau_{\omega avrg}$ and $\tau_{\omega max}$ at a flow velocity of 100 µm/s:

(6) $$\tau_{\omega avrg}=9.82\cdot 10^{-12}k^{-0.914}$$

(7) $$\tau_{\omega max}=3.36\cdot 10^{-10}k^{-0.807}$$

In fact, Equations (5)–(7) are only valid in systems with laminar flow assuming Darcy’s law is applicable. This is the case as long as the interstitial Reynolds number *Re_i_* < 8 [28]. Consequently, *Re_i_* was calculated from the bioreactor and scaffold parameters:

(8) $$Re_{i}=\frac{\rho \psi uD_{P}}{µ\left(1-\varepsilon \right)}$$

where *ρ* is the density of water at 37 °C, *D_P_* is the average pore diameter, *ψ* is the sphericity (for simplicity assumed to be 1), and *ε* is the porosity.

### 2.8. Cell Culture

Human ASCs used in this study were isolated from female donors (42, 48, and 52 years old) as described previously [24]. Isolation from human tissue was approved by the ethics committee of the Medical University Vienna, Austria (EK Nr. 957/2011, date: 30 January 2013). All donors gave written consent. Briefly, fat tissue obtained from abdominoplasty was minced with scissors and digested with collagenase type I (Sigma Aldrich, St. Louis, MO, USA). After several centrifugation and washing steps, the stromal vascular fracture was released in a cell culture flask and ASCs were selected by plastic adherence. The donor tissue was processed within 3–6 h after surgery.

After isolation, ASCs were cultivated in standard medium composed of MEM alpha (Thermo Fisher Scientific, Waltham, MA, USA), 0.5% gentamycin (Lonza, Basel, Switzerland), 2.5% human platelet lysate (PL BioScience, Aachen, Germany), and 1 U/mL heparin (Ratiopharm, Ulm, Germany) in a humidified incubator at 37 °C and 5% CO_2_. Cells were cryo-preserved in 77.5% αMEM, 12.5% HPL, 10% DMSO (Sigma Aldrich), and 1 U/mL heparin, as described previously [9]. After thawing, the cells were expanded for two passages in T-flasks (Sarstedt, Nümbrecht, Germany) and harvested via accutase (GE healthcare, Little Chalfont, UK) treatment to be used for cultivation in the bioreactor system. For cell culture experiments, cells of the three donors were mixed in equal amounts before seeding.

### 2.9. Bioreactor Cultivation

To evaluate the influence of the different flow profiles and shear stress distributions of BR1 and BR2 on osteogenic differentiation, human ASCs were cultivated for 21 days on Sponceram. Prior to seeding, Sponceram matrices were steam sterilized. After steam sterilization, Sponceram matrices were seeded with 50 µL of a 6 × 10^6^ cells/mL cell suspension of ASCs (passage 2, *n* = 3 donors) and incubated for 2 h at 37 °C before they were carefully covered with medium. Cells seeded on a 12 well plate (4000 cells/cm^2^) served as the 2D static control. The seeded matrices were transferred to the bioreactor chamber (3D dynamic) after 3 days or were kept in the well of a 6 well plate (3D static). The bioreactors and 6 well plates were filled with either 10 mL osteogenic differentiation medium (ODM; standard medium supplemented with 5 mM beta-glycerolphosphate, 0.1 μM Dexamethasone, 200 µM l-ascorbate-2-phosphate, all from Sigma Aldrich, St. Louis, MI, USA) whereas the 12 well plates were filled with 2 mL. Perfusion in the bioreactors were set to a flow rate of 1.5 mL/min. Cells were cultivated for 21 days and the medium was changed every 2–3 days (1 mL for determination of ALP activity, glucose, and lactate) while 6 mL were exchanged on day 7 and 14.

### 2.10. DNA Quantification

Prior to DNA extraction, 3D samples were grinded whereas 2D samples were detached by accutase treatment. Lysis buffer containing 0.1 mg/mL Proteinase K (Sigma Aldrich) was then added to each sample before incubating the samples (3 h, 37 °C, 100 rpm). DNA was precipitated with 100% ethanol and after centrifugation (14,000× *g*, 20 min, 4 °C) was washed with 70% ethanol. The pellet was dried, resuspended in TE buffer, and stored at 4°C. DNA was quantified using the Invitrogen™ Quant-It™ PicoGreen^®^ dsDNA Assay Kit according to instructions provided by the manufacturer (Invitrogen, Carlsbad, CA, USA).

### 2.11. Alkaline Phosphatase Activity

Alkaline phosphatase (ALP) as an osteogenic marker can be detected in the cell culture supernatant. To determine the ALP activity, the supernatants were transferred into a 96-well plate (8 × 80 µL per condition) and 20 µL of a p-nitrophenyl phosphate stock solution (Sigma Aldrich) was added to each well. After 60 min of incubation, the absorption at 405 nm was detected and the ALP activity was calculated from this data.

### 2.12. DAPI Staining

Prior to staining the cell nuclei with 4′,6-diamidin-2-phenylindol (DAPI), the samples were fixated with 96% ethanol. Cells or scaffolds were rinsed with PBS, covered with DAPI staining solution (1 µL DAPI stock in DAPI buffer), and incubated for 20 min at room temperature. Subsequently, the cells were rinsed with PBS twice and documented by fluorescence microscopy (excitation/suppression filter: 360/470 nm). For better comparability, the fluoresence micrographs of the different conditions were taken with consistent parameters (exposure, gain, and gamma) and images of the entire sample were acquired at 4-fold magnification (30–40 images per sample). Subesquently, images were digitally merged with the software “Microsoft Image Compositor Editor” to give a comprehensive overview of the entire scaffold.

### 2.13. Matrix Mineralization

Calcium content of the extracellular matrix was observed by calcein (calcium deposition) and von Kossa (phosphate deposition) stain. Samples were fixated with 96% ethanol. For calcein staining, samples were washed with ddH_2_O and incubated over night at 4 °C in calcein staining solution (0.1 µg/mL in ddH_2_O; Sigma Aldrich). Afterwards, cells were washed with PBS and observed with a fluorescence microscope (exposure: 205 ms; gain: 1×; gamma: 1×).

To observe phosphate deposition, the fixated cells were washed with ddH_2_O and incubated in 1 mL AgNO_3_ solution (5% *w*/*v*; Carl Roth) for 30 min and were light protected. After the samples were rinsed with ddH_2_O and exposed to UV light for 2 min (each side), they were incubated in Na_2_S_2_O_3_ (5% *w*/*v*, Sigma Aldrich). Samples were rinsed with ddH_2_O and the staining was documented with a flatbed scanner.

### 2.14. Statistical Analysis

All data are expressed as mean values ± standard deviation. The data was analyzed using Microsoft Excel, OriginPro, and GraphPad Prism. Multiple comparisons were carried out using one-way analysis of variance followed by the Dunnett’s or Tukey’s multiple comparisons test. Values of *p* < 0.01 with a confidence interval of 99% were defined as statistically significant.

## 3. Results

### 3.1. Flow Profile and Residence Time Distribution

**Figure 4:**
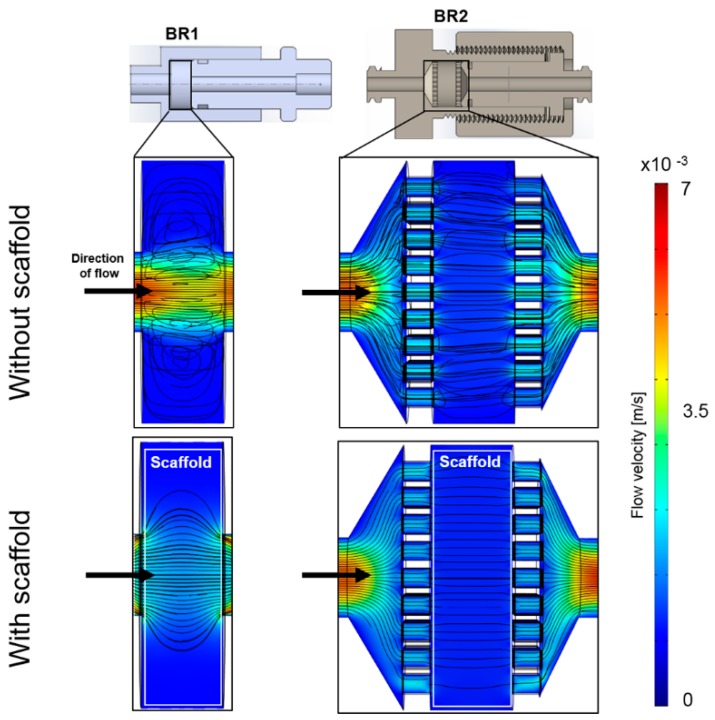
Computational model of the flow profile with streamlines of the bioreactor chambers BR1 and BR2 without and with a scaffold inserted (porosity: 66.7%, permeability 1.74 × 10^−10^ m^2^) at a flow rate of 1.5 mL/min (3.6 mm/s). Streamlines and the flow profile indicate a more homogeneous flow distribution in BR2 due to the medium distribution units.

A homogeneous flow profile throughout the bioreactor chamber is preferable since nutrition supply and waste removal is crucial in every 3D cultivation. Thus, a computational study was conducted to simulate the distribution of flow velocity and streamlines in the bioreactor with and without a scaffold inserted. The flow profile of the empty BR1 indicates a higher flow velocity exclusively in the center of the chamber (Figure 4). In contrast, the outer areas display velocities close to zero while circular streamlines indicate dead spaces. After introducing the scaffold into the model, higher flow velocities were especially observed in the center region. In contrast, the medium distribution units (MDUs) of BR2 seem to support a homogeneous flow profile. The average and maximum velocity of the fluid that passed through the scaffold was derived from the CFD data (Table 1). In BR1 the maximum flow velocity is 16-fold higher than the average velocity whereas it is only 5-fold higher in BR2. Therefore, the MDUs seem to support a homogeneous flow velocity profile and reduce flow velocity peaks.

**Figure 5:**
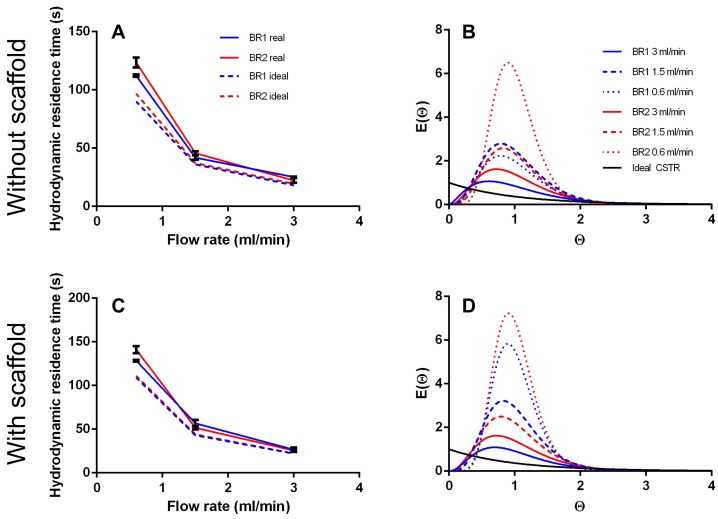
(**A**) Hydrodynamic residence time and (**B**) residence time distribution of BR1 and BR2 without inserted scaffold. (**C**) Hydrodynamic residence time and (**D**) residence time distribution of BR1 and BR2 with inserted scaffold.

The residence time distribution (RTD) of a bioreactor characterizes the mixing behavior, and consequently is an important parameter in bioprocess engineering. In this study we compared the RTDs of two different bioreactor chambers at different flow rates with or without a scaffold inserted. Ratios of the ideal hydrodynamic residence time *T* and the real mean residence time *T_m_* at different flow rates were compared (Table 2, Figure 5).

*T* is a theoretical value that describes the time a fluid volume needs to pass through the bioreactor when no back mixing occurs (like in an ideal PFR). As expected, both bioreactors did not behave like an ideal PFR and *T_m_* was higher than *T* (12–37%), indicating back mixing. Moreover, *T_m_*/*T* does not appear to be affected by flow rate or bioreactor geometry significantly. However, in BR2 the ratio of *T_m_*/*T* seems to decrease with higher flow rates and is lower at 3 mL/min than in BR1 showing a more PFR like behavior.

The curve fit of the RTDs of the empty bioreactor indicate a correlation between increasing flow rate and stronger mixing, resulting in a more CSTR like mixing behavior (Figure 5). At lower flow rates the fluid behaves more like that in a PFR. Besides, inserting a scaffold seems to inhibit back mixing and instead promotes a more uniform flow.

The tanks in series and the Bodenstein number *Bo* were both derived from the RTDs and are compared in Table 3. Generally, more tanks in series were observed at lower flow rates indicating a more plug flow like behavior. The chamber geometry of BR2 seems to support a more laminar flow with less back mixing whereas the chamber of BR1 seems to improve back mixing. Again, the insertion of a scaffold into the fluid pathway supports a plug flow behavior. For the flow rates of 1.5 and 3.0 mL/min, *N* is between 2.5 and 5.7. When *N*→1 the system is considered to be mixed completely. Thus, a flow rate of 1.5 mL/min and higher can be considered as optimal mixing.

The Bodenstein number was derived from the raw data (not from the global curve fit) and should behave similar to the number of tanks-in-series model. It is highest at the lowest flow rate indicating low axial dispersion (except for BR1 empty). Although, *Bo* does not decrease with the flow rate considerably like the number of tanks in series.

The data from the computational model together with the RTDs indicate almost optimal mixing in BR1 with a heterogeneous flow profile. In contrast, BR2 demonstrated less mixing but a homogeneous flow velocity profile.

### 3.2. Sensor Characterization and Determination of Permeability

**Figure 6:**
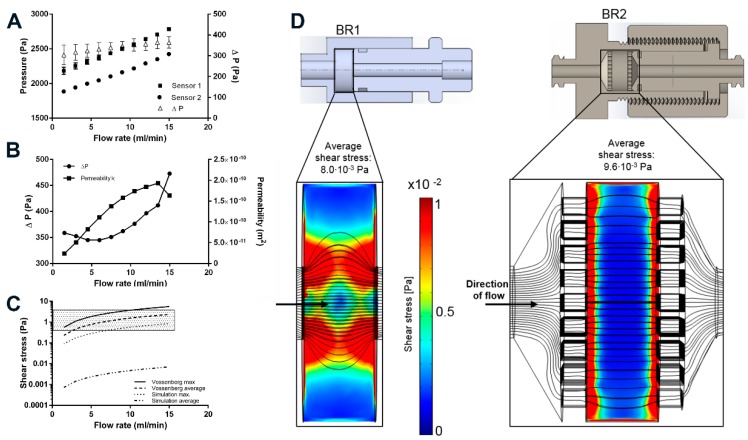
(**A**) Sensor characterization at different flow rates. Δ*P* depicts the differential of both sensors; (**B**) Determination of the permeability of the porous scaffold Sponceram. Δ*P* and the resulting permeability k at different flow rates; (**C**) Shear stress prediction based on the determination of the permeability of Sponceram and further calculation with the Vossenberg model or CFD data. Depending on the flow rate, the analytical model predicts shear forces in the physiological in vivo range of 0.3–3 Pa (shaded area) whereas the computational modeling predicts shear forces that are approximately three orders of magnitude lower; (**D**) Computational simulation of the shear stress conditions. BR1 and BR2 with porous scaffold (porosity: 66.7%, specific permeability 1.74 × 10^−10^ m^2^) at a flow rate of 15 mL/min. The model indicates a more homogeneous distribution of shear stress in BR2.

The installed pressure sensors are usually used for medical purposes to monitor the blood pressure of patients in intensive care units by piezoresistive transducers. According to the manufacturer’s specifications, the sensors together with the amplifier have a pressure range of −20 to 300 mmHG (−2.6–40 kPa). Indeed, flow rates used in 3D perfusion cell culture are often as low as 0.3–3 mL/min [29], resulting in a flow induced pressure of approximately <3 kPa (depending on the tubing diameter). Hence, these implemented sensors may not measure as accurately as in their original setting. Still, the characterization of the sensors show a strong linear correlation of pressure and flow rate (R^2^ > 0.997, *p* < 0.0001; Figure 6). Interestingly, the standard deviation of the measurements decreased with increasing flow rate indicating a more accurate measurement at higher flow rates. The pressure differential of both sensors was found to be very constant at 338 ± 20 Pa (2.5 ± 0.5 mmHg).

To determine the permeability *k* of Sponceram, the pressure differential upstream and downstream of the bioreactor was recorded at different flow rates. Since *k* is a material constant, Δ*P* should increase proportionately with the flow rate. Although the pressure sensors appear to be accurate, the calculated permeability changed with the flow rate (Figure 6). Δ*P* increased linearly for flow rates >7.5 mL/min while *k* remained almost stable. Therefore, the average of the calculated *k*-values from 9 to 15 mL/min (*k* = 1.7 ± 0.9 × 10^−10^ m^2^) was inserted in the shear stress equations and the computational model. Similarly, the permeability of Sponceram with a higher porosity (80%) than the Sponceram discs used in this study was found to be *k* = 1.88 × 10^−8^ m^2^ and the permeability of cancellous bone was approximately 2.1 × 10^−9^ m^2^ as reported before [25]. The lower permeability measured in the present study can be attributed to the lower porosity (67%) of Sponceram used in this study.

### 3.3. Fluid Shear Stress Estimation

Mechanical stimuli such as shear forces are commonly known to influence cellular behavior. Fluid shear forces can induce the differentiation of stem cells towards specific lineages which is important in all dynamic cell culture processes [30]. In this study we conducted a computational simulation to compare the shear stress distribution in two different bioreactor chambers on a bone like scaffold. The interstitial Reynolds number *Re_i_* was calculated first to ensure that Darcy’s law is applicable (*Re_i_* < 8). *Re_i_* was found to be between 0.28 and 7 for flow rates between 1.5 and 15 mL/min, thus indicating a laminar flow. Depending on the flow rate and bioreactor geometry, the average shear stress calculated by the simulation was between 0.1 × 10^−2^ and 1 × 10^−2^ Pa, and the maximum shear stress was between 8.8 × 10^−2^ and 85.2 × 10^−2^ Pa (Table 4). Although the average shear stress is similar in both bioreactor models, the maximum shear stress is about 2-fold higher in BR1, indicating higher stress peaks due to the bioreactor geometry.

Generally, the results of the simulation indicate higher shear forces at the surfaces of the scaffold where the fluid enters. In BR1, higher shear forces occur at the entrance, core, and exit of the scaffold whereas the outer areas show very low shear forces. In contrast, BR2 displays a much more homogeneous shear stress distribution throughout the scaffold (Figure 6).

Furthermore, an analytical model that uses the permeability *k* as an indicator for wall shear stress was used to estimate shear stress and was compared to the CFD derived calculation. The Vossenberg model predicts average shear stress between 0.23 and 2.34 Pa which is approximately 3 orders of magnitude lower than that predicted by the CFD simulation. Maximum shear forces were calculated to be between 0.56 and 5.6 Pa which is approximately 6-fold higher than that estimated by the CFD simulation (Figure 6). Simplification of the scaffold in the computational model probably lowers the prediction of shear forces. Also, in a study by Jungreuthmayer, et al. [31] where µCT data of a scaffold was used for CFD analysis, the computational model underestimated shear forces compared to the analytical models.

Although estimations from computational and analytical models differ from each other, the shear forces calculated from the analytical models were in the range of in vivo shear stress of bone, which is expected to be between 0.3 and 3 Pa [32]. Therefore, this bioreactor-incubator system, together with this scaffold, may represent a suitable system for bone tissue engineering.

### 3.4. Bioreactor Cultivation

**Figure 7:**
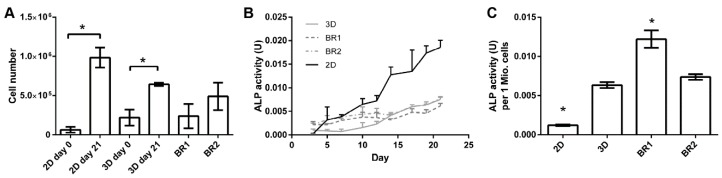
(**A**) Cell numbers, (**B**) course of alkaline phosphatase (ALP) activity and (**C**) ALP activity per cell of adipose derived mesenchymal stem cells (ASCs) cultivated on Sponceram in a perfusion bioreactor or under 3D and 2D static conditions. Data are represented as mean ± SD (*n* = 3); * significant difference of the indicated conditions in panel (**A**) or to 3D static in panel (**C**) with a confidence interval of 99% and *p* < 0.001.

**Figure 8:**
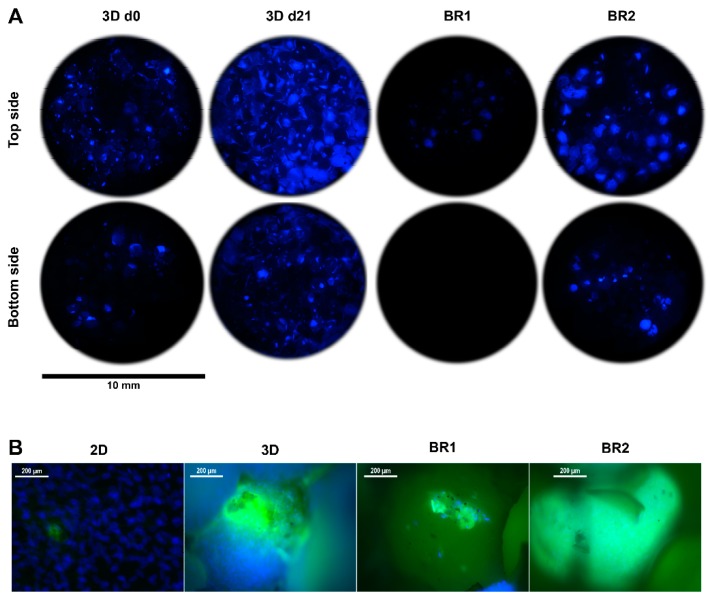
(**A**) 4′,6-diamidin-2-phenylindol (DAPI) stain and (**B**) DAPI-calcein double stain of Sponceram after 21 days of cultivation with ASCs in a perfusion bioreactor or under static conditions.

To investigate the influence of the different shear stress distributions and flow profiles of BR1 and BR2 on osteogenic differentiation of human ASCs, cells were cultivated for 21 days in ODM on the 3D ceramic matrix Sponceram. Seeded matrices in 6 well plates (3D static) and cells seeded in conventional 12 well plates (2D static) served as controls. The cell number of each sample was determined indirectly via DNA quantification and was highest in the 3D and 2D control group. However, twice as many cells were found in BR2 compared to BR1 where the cell number did not change significantly compared to day 0 (Figure 7). These findings were also confirmed by DAPI staining (Figure 8).

**Figure 9:**
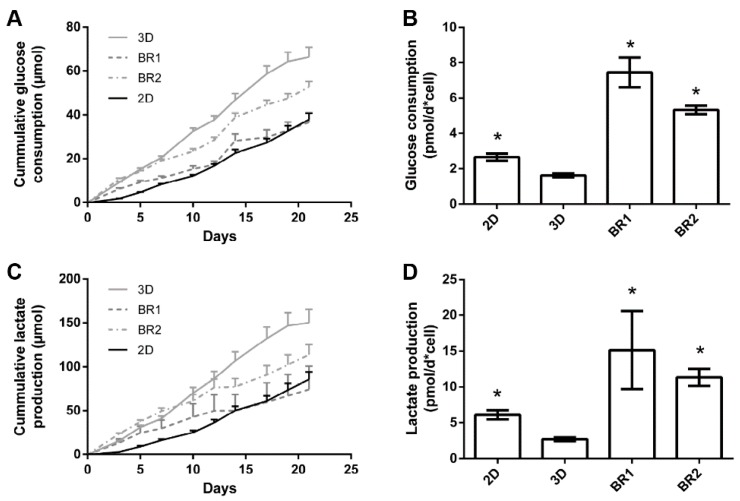
Course of (**A**) glucose consumption and (**C**) lactate production, (**B**) glucose consumption and (**D**) lactate production per cell between day 19 and 21 of ASCs cultivated on Sponceram in a perfusion bioreactor or under 3D and 2D static conditions. Data are represented as mean ± SD (*n* = 3); * indicates significant difference to 3D static with a confidence interval of 99% and *p* < 0.001.

Glucose consumption and lactate production were steady throughout the entire cultivation period although lactate production decreased slightly after 12 days in dynamic conditions (Figure 9). The overall glucose consumption and lactate production was found to be highest in 3D static conditions. However, consumption and production per cell between day 19 and 21 indicates a higher glycolytic activity in cells cultivated in dynamic conditions (Table 5).

ALP activity, a marker for osteogenic differentiation, increased after approximately 12–14 days in 2D and 3D static conditions. In contrast, in dynamic conditions it was elevated from day 3 on but did not increase as much as under static conditions after 14 days. However, ALP activity per cell revealed an increased activity in all 3D conditions compared to 2D conditions, while the activity per cell in BR1 was found to be higher compared to BR2 (Figure 7).

**Figure 10:**
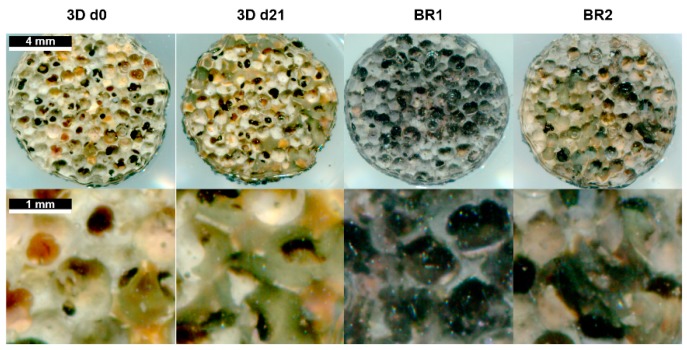
Von Kossa stain of Sponceram after 21 days of cultivation with ASCs in a perfusion bioreactor or under static conditions.

Matrix mineralization was determined by calcein and von Kossa stain. Calcium depositions were found in all conditions (Figure 8 and Figure 10). However, only few stained areas were observed. Furthermore, phosphate depositions were found to be increased in BR1 but only slightly in BR2 and 3D static conditions.

## 4. Discussion

In this study we developed a miniaturized perfusion bioreactor system together with a specialized incubator system for use in different cell culture applications and for bioprocess optimization. At first, the incubator system was developed and modified to serve as a flexible platform for bioreactor cultivation. For this, it was equipped with pressure sensors, hydrostatic pressure valves, and multi-channel pumps that can be programmed according to the user’s needs.

Next, in order to establish a flexible perfusion system we designed, manufactured, characterized, and compared two different bioreactor chambers. The general advantages of both chambers are depicted in Table 6. Hence, the flow velocity profiles of both the bioreactors were simulated and the mixing behavior was characterized by measuring the residence time distribution. Though, BR2 displayed a more PFR like mixing behavior compared to BR1, flow rates of 1.5 mL/min and higher were considered to provide sufficient mixing. Although the RTDs from both bioreactors did not differ considerably, the flow field experienced by cells might be different [33]. Recirculation areas found in the CFD simulation of BR1 indicate a different flow field than in BR2 where MDUs prevent those recirculation areas. The tanks-in-series model also revealed less axial dispersion in BR2. Indeed, MDUs in BR2 promote a uniform flow velocity profile and plug flow like behavior throughout the scaffold while preserving proper mixing.

Furthermore, the permeability of a porous scaffold was determined with pressure sensors in order to estimate shear stress with the model proposed by Vossenberg and a CFD simulation. Medium distribution units of BR2 proved to be beneficial in promoting a uniform flow velocity and shear stress distribution throughout the scaffold. A homogeneous flow and shear stress profile is highly preferable since this ensures maximum control and reproducible results during the experiments. To investigate effects of the different flow and shear stress profiles, both bioreactor chambers were compared in a proof-of-concept bioreactor cultivation study.

For this, the flow rate was chosen according to the calculations of the simulation and mathematical model as follows. The average shear stress at a flow rate of 1.5 mL/min was between 0.001 (CFD) and 0.23 Pa (Vossenberg) and the maximum shear stress was between 0.08 and 0.56 Pa which only partially lies in the physiologic in vivo shear stress of 0.3–3 Pa. However, osteogenic differentiation performed in 3D conditions was found to be increased with shear stress by about one order of magnitude lower than the in vivo shear stress [6]. Seemingly, lower shear forces are sufficient to induce osteogenic differentiation. Also, the maximum shear stress was shown to increase dramatically compared to the average shear stress with increasing flow rate if the permeability constant *k* is below 1·× 10^−10^ m^2^ [27]. In the present work, the permeability constant *k* of the scaffold was found to be 1.7 ± 0.9 × 10^−10^ m^2^. Furthermore, the Vossenberg model was developed for scaffolds with perpendicular fibers and thus the permeability might not be as predictive for shear stress as in a porous scaffold. Also, the CFD simulation lacks accuracy since it is based on a simplified geometry of the scaffold. Taken all together, the flow rate of the proof of concept study was set to 1.5 mL/min to generate shear forces at the lower end of the physiologic range in order to avoid excessive washout of cells.

After 21 days of cultivation, the cell number was found to be increased in BR2 compared to BR1 while the cell distribution was more homogeneous as indicated by DAPI staining. However, cell number and glucose consumption indicate lower proliferation in dynamic conditions compared to static conditions. Thus, cells in dynamic conditions might have been subject to washout by perfusion. The ratio of lactate production per mole consumed glucose is commonly used as an index for anaerobic metabolism which occurs mainly in proliferating mesenchymal stem cells. These cells display a higher ratio since they generate energy rather by anaerobic glycolysis than by oxidative phosphorylation [34]. Indeed, cells cultivated under dynamic conditions display a lower ratio (2.02–2.15) than under static conditions (2.26) which might indicate a shift to oxidative phosphorylation and therefore to differentiation. Also, ALP activity per cell was found to be higher in dynamic conditions and phosphate deposition was only visible in BR1. These findings suggest increased differentiation in dynamic conditions. However, the glucose consumption and lactated production per cell was found to be significantly higher in dynamic conditions compared to static conditions. Controversially, a previous study by Pattappa et al. [35] reported reduced glycolytic activity during osteogenic differentiation. However, this study was carried out in conventional 2D static conditions. In contrast, 3D dynamic conditions increase the mass transfer of nutrients, oxygen, and waste products, which together with mechanical stimulation might alter metabolic activity in comparison to 2D static cultivation conditions.

Regarding the influence of the flow and shear stress profile of BR1 and BR2 on the osteogenic differentiation, cells of BR1 showed increased ALP activity per cell and matrix mineralization compared to BR2. Interestingly, phosphate depositions were present only in the center of BR1 where flow velocity and shear stress is the highest as indicated by the CFD simulations. Furthermore, the maximum flow velocity at 1.5 mL was found to be 3.6-fold higher in BR1. Although it did not affect the average shear stress, it caused a 1.9-fold increase in the maximum shear stress. Also, the maximum shear stress in BR1 was 88-fold higher than the average shear stress while it was only 46-fold higher in BR2. The findings of the CFD simulation together with the data from bioreactor cultivation suggest that in order to generate shear stress as high as in BR1, the volumetric flow rate needs to be increased in BR2. In this study, where both bioreactor chambers were operated at the same flow rate, BR1 supported an increased osteogenic differentiation of ASCs while BR2 maintained homogeneous cell growth rather than differentiation. Since it is likely that differentiation was induced by higher shear stress in the center of BR1, a higher flow rate resulting in similar shear stress conditions might foster comparable differentiation in BR2 as well. In conclusion, depending on the mathematical model, shear stress calculations derived from characterization of the matrix and CFD simulation suggested physiologic shear stress conditions for a broad flow rate spectrum of 1.5 to 15 mL/min. However, simulations were predictable for the relative comparison of BR1 and BR2, and an optimal flow rate for the generation of physiologic shear stress still needs to be evaluated by experiments. The successful screening of different shear stress and hydrostatic pressure conditions in this system was demonstrated before on a decellularized bone matrix [23].

Perfusion systems have been proven to be beneficial for tissue engineering purposes such as bone or cartilage engineering [36,37]. For instance, numerous studies show a higher matrix deposition or upregulation of relevant genes in comparison to static culture conditions when bone precursor cells are exposed to fluid shear stress [38,39,40,41,42,43]. Although several tailor-made [10,11,44] and commercially available [45,46,47] perfusion systems were developed and successfully used, there is a lack of automated sensor-controlled systems that allow the cultivation of multiple independent replicates under different conditions.

However, the presented system allows the determination of the permeability of scaffolds, thus enabling estimation and control of shear stress during cultivation. Since different flow and pressure regimes can be programmed with the built-in control unit, the incubator allows comprehensive control over the mechanical environment. The bioreactor chamber developed in this study has been used with hard scaffolds (e.g., ceramics) but was designed to host also soft scaffolds (e.g., hydrogels) of different sizes via integrated grids. Together with the possibility to cultivate up to sixteen independent reactors (with an upgrade of the pumping head) in one incubator, we hypothesize it can be used not only in the field of bone tissue engineering but also in a wider variety of 3D cell culture processes. Further studies will focus on demonstrating its benefit in the testing and optimization of different dynamic cell culture conditions (including hydrostatic pressure) on other biomaterials.

## 5. Conclusions

A parallelizable, miniaturized perfusion bioreactor system, together with a tailor-made incubator, was developed and characterized. In particular, it was designed to be flexible and modular in terms of exchanging different parts such as the tubing, pumping head, and scaffold or adding additional instrumentation, such as pinch valves for the application of hydrostatic pressure. Integrated pressure sensors allow the estimation of fluid shear stress that cells experience on a scaffold and as a result permit the screening of the effects of different mechanical culture conditions. The effect of different flow and shear stress profiles were investigated in a proof of concept study. CFD data and shear stress calculations were found to be predictable for a relative comparison of two bioreactor geometries, but not for the prediction of the optimal flow rate. We assume a parallelizable miniaturized system where only small amounts of cells, culture medium, and scaffolds are required, that will support throughput and reproducibility, and thus will be beneficial for the optimization of dynamic 3D cell culture applications.

## Figures and Tables

**Table 1:** CFD derived data of the average and maximum velocities of fluid passing the scaffold inside the bioreactor chambers BR1 and BR2.

Bioreactor	Inlet Velocity [mm/s]	Average Velocity [mm/s]	Maximum Velocity [mm/s]	Maximum/Average Velocity
BR1	3.5	0.4	6.2	16.0
	17.7	1.9	30.0	15.4
	35.4	3.9	56.5	14.5
BR2	3.5	0.3	1.7	5.3
	17.7	1.6	8.6	5.3
	35.4	3.2	17.3	5.4

**Table 2:** Differences of the mean residence time *Tm* to the ideal hydrodynamic residence time *T* in percent (at least *n* = 3).

Condition	Difference to *T* [%]		
Flow rate (mL/min)	0.6	1.5	3.0
BR1 empty	16 ± 0.1	12 ± 0.4	30 ± 0.3
BR2 empty	37 ± 1.1	27 ± 0.8	23 ± 1.7
BR1 with scaffold	18 ± 0.1	31 ± 1.7	23 ± 1.7
BR2 with scaffold	27 ± 0.6	16 ± 0.5	15 ± 0.7

**Table 3:** Tanks in series and Bodenstein number of both bioreactors with and without the scaffold. Tanks in series are derived from a global curve fit (*n* = 3). The Bodenstein number was calculated from the raw data set.

Flow Rate (mL/min)	Tanks in Series			Bodenstein Number		
	0.6	1.5	3.0	0.6	1.5	3.0
BR1 empty	4.6	4.8	2.5	6.1	6.3	7.0
BR2 empty	9.7	4.8	2.5	7.4	6.7	5.9
BR1 with scaffold	9.7	5.7	3.3	8.0	7.3	7.7
BR2 with scaffold	10.7	4.7	3.4	9.1	7.2	7.6

**Table 4:** Data from the CFD simulation and Vossenberg model describing the average and maximum shear stress inside the scaffold.

Bioreactor	Inlet Velocity [mm/s]	CFD Simulation		Vossenberg Model	
		Average Shear Stress [10^−2^ Pa]	Maximum Shear Stress [10^−2^ Pa]	Average Shear Stress [10^−2^ Pa]	Maximum Shear Stress [10^−2^ Pa]
BR1	3.5	0.1	8.8	23.4	56.0
	17.7	0.4	43.4	117.2	280.0
	35.4	0.8	85.3	234.4	560.0
BR2	3.5	0.1	4.6	-	-
	17.7	0.5	22.9	-	-
	35.4	1.0	45.4	-	-

**Table 5:** Glucose consumption and lactate production of ASCs under different cultivation conditions (*n* = 3).

Condition	Cumulative Glucose Consumption (µmol)	Cumulative Lactate Consumption (µmol)	Ratio
2D	37.9 ± 2.9	85.5 ± 8.7	2.26
3D	66.3 ± 4.5	150.1 ± 15.0	2.26
BR1	36.6 ± 4.1	74.2 ± 26.7	2.15
BR2	52.8 ± 2.4	113.6 ± 11.7	2.02

**Table 6:** Conclusive overview on the characteristics of BR1 and BR2.

Aspect	BR1	BR2
Advantages	- Sufficient mixing - Increased matrix mineralization - Increased ALP activity/cell	- Sufficient mixing - Homogeneous flow profile and shear stress distribution throughout the scaffold - Increased proliferation - More homogeneous growth on the scaffold
Disadvantages	- Inhomogeneous flow profile and shear stress distribution	- Comparably low matrix mineralization
